# Fuzzy logic–based clinical decision support system for the evaluation of renal function in post‐Transplant Patients

**DOI:** 10.1111/jep.13302

**Published:** 2019-11-12

**Authors:** Giovanni Improta, Valeria Mazzella, Donatella Vecchione, Stefania Santini, Maria Triassi

**Affiliations:** ^1^ Department of Public Health of the University Hospital University of Naples Federico II Naples Italy; ^2^ Department of Electronic Engineering and Information Technology, Faculty of Engineering University of Naples Federico II Naples Italy

**Keywords:** clinical decision support systems, fuzzy logic, health care decision problems

## Abstract

**Objectives:**

In the context of the gradual development of artificial intelligence in health care, the clinical decision support systems (CDSS) play an increasing crucial role in improving the quality of the therapeutic and diagnostic efficiency in health care. The fuzzy logic (FL) provides an effective means for dealing with uncertainties in the health decision‐making process; therefore, FL‐based CDSS becomes a very powerful tool for data and knowledge management, being able to think like an expert clinician. This work proposes an FL‐based CDSS for the evaluation of renal function in posttransplant patients.

**Method:**

Based on the data provided by the Department of Nephrology of the University Hospital Federico II of Naples, a statistical sample is selected according to appropriate inclusion criteria. Four fuzzy inference systems are implemented monitoring the renal function by the level of proteinuria and the glomerular filtration rate (GFR).

**Results:**

The systems show an accuracy of more than 90% and the outputs are provided through easy to read graphics, so that physicians can intuitively monitor the patient's clinical status, with the objective to improve drugs dosage and reduce medication errors.

**Conclusions:**

We propose that the CDSSs for the assessment and follow‐up of kidney‐transplanted patients built in this study are applicable to clinical practice.

## 1 INTRODUCTION

The computer science is able to help clinicians in the formulation of a diagnosis in order to make the correct decisions for therapeutic purposes and for the prediction of outcome, becoming an indispensable support for modern medicine to solve complex clinical problems too.^1^ Among the most widely used IT tools in the medical field, there are the clinical decision support systems (CDSS), defined as computer programmes assisting physicians in making decisions, exhibiting sophisticated reasoning capabilities in order to improve clinical decision making, thus promoting more efficient care practices.^2^ Early CDSSs are designed by researchers on expert systems, with the aim of programming the computers with rules that allow it to “think” like an expert clinician when it compared with a patient.^3^ The CDSSs provide several modes of decision support such as critical values alerts, advice for drug prescribing, comments on existing health care orders, and suggestions for various active care issues.^4^ In literature, different decision‐making support tools can be found, and most of them were based on IT programmes and algorithms to analyse and elaborate biomedical data and signals^5^, ^6^, ^7^, ^8^, ^9^, ^10^; some of them can also be based on multicriteria decision‐making methods, like analytic hierarchy process, or can be even used in combination with management approaches, like Lean and Six Sigma, finding applications in different areas like health technology assessment,^11^, re‐engineering of health care processes,^12^, ^13^ and improvement of clinical outcomes or patient satisfaction.^14^, ^15^, ^16^, ^17^

Since the 1950s, there have been multiple attempts to construct a programme that would assist clinicians with their decisions concerning diagnosis and therapy. Ledley and Lusted published a pionieristic work evolving around this idea.^18^ However, the first operative CDSS did not appear until the 1970s, when de Dombal et al^19^ developed “Leeds Abdominal Pain System,” using Bayesian reasoning on the basis of surgical and pathological diagnoses.^19^ In the 1990s, the so called “HELP system” was perfected to integrate the Hospital Information System (HIS) with the ability to generate alerts when data abnormalities in the patient record are noticed. Along with this line, different systems are currently in use.^20^ For example, in the emergency department (ED) of Saudi Arabian hospitals, a CDSS to deal with acute coronary syndrome (ACS) was implemented to give to emergency physicians the ability to improve their practices, to assist them in dealing with life threatening diseases, and to provide accurate decisions in real time in order to save a patient's life.^21^

The standards of judgement of a CDSS are widely reported in the literature. Among them, the most important are the improving patient safety through reduced medication errors and test ordering, improving quality of care by increasing available time for direct patient care, and improving efficiency in health care delivery by reducing costs and test duplication.^22^

A characterizing element of a CDSS is the knowledge, which allows the system to provide specific information on filtered patients in an intelligent way, in order to improve their effectiveness. Indeed, there are two types of CDSS: knowledge‐based and non‐knowledge–based CDSS. The latter uses the artificial intelligence principles to machine learning through neural networks or genetic algorithms.

Among the knowledge‐based systems, there are the fuzzy logic (FL)‐based CDSS. These systems are based on rules mostly in the form of if‐then statements, and the data are usually associated with these rules.^23^

The use of FL‐based CDSS in health systems has spread successfully in recent years by investing almost all medical fields, following some examples of FL‐based CDSS are reported. Soesanti et al^24^ presented a FL method for magnetic resonance imaging (MRI) brain images segmentation. Sizilio et al^25^ developed a fuzzy method that provided breast cancer prediagnosis with 98.59% sensitivity where the prediction of the risk is based on a set of judiciously chosen fuzzy rules that utilized patient age and tumour features. A fuzzy mathematical model of HIV infection consisting of a linear fuzzy differential equations system is used by Zarei at al^26^ to describe the ambiguous immune cell level and the viral load that was due to the fuzziness behaviour of the immune system in HIV‐infected patients. In the pharmacy field, Mauselth et al^27^ used fully automated FL‐based closed‐loop insulin dosing controller that allows clinicians to personalize patients dosing. In risk classification of renal diseases using FL‐based CDSS, the works of Ahmed and Narasimhan are of interest. The work of Ahmed et al is interesting for diagnostic report of the healthiness of a patient's kidney used as a following input variables set: nephron functionality, blood sugar, blood pressure, age, weight, and alcohol intake.^28^ The second for risk classification of diabetic nephropathy with following input parameters: plasma glucose concentration, diastolic blood pressure, body mass index, and age. 29 More recently, Santini et al^3^ developed a fuzzy inference machines to improve the knowledge‐based CDSS actually used in the day‐by‐day clinical care of β‐thalassemia patients of the Rare Red Blood Cell Disease Unit (RRBCDU) at “AORN A. Cardarelli” Hospital in Naples. This work shows exemplary results on the online evaluation of iron overload during the health status assessment and care management of β‐thalassemia patients.^3^

CDSS can be used as an effective tool in order to reduce morbidity and mortality rates in patients with renal failures. Indeed, after renal transplantation, several complications may arise that result in a serious impaired renal function.^30^ These dysfunctions may not only appear very early after transplantation (as early as in the operating room) but may also arise very late (months later).^31^

Furthermore, some of the complications may also result in deterioration of renal function as a late permanent event and, hence, very careful monitoring of patients is required to detect complications before severe damage happen.^30^

Among the risks resulting from a renal transplant, the most important is certainly rejection, evadable by using immunosuppressant drugs that have the purpose of controlling the activity of the immune system. However, these drugs have significant side effects that can seriously worsen the living conditions of the transplanted. Keeping a balance between the effective prevention of rejection and the side effects of immunosuppressant drugs is a key point for long‐term renal transplantation success.^32^ Moreover, maintaining this balance is made even more complex in diabetic patients because hyperglycaemia causes the excess of glucose that over time forms irreversible end‐products; the tissue accumulation of these products contributes to the associated renal and microvascular complications.^33^

Another consequence of a transplant is hypertension that should be treated with ACE inhibitor drugs. In addition to blunting the hypotensive effects, these drugs increase the risk of acute renal failure especially when an NSAID (nonsteroidal anti‐inflammatory drugs) is coadministered, leading to increased serum creatinine concentrations and GFR (glomerular filtration rate) decrease. Furthermore, these drugs can reduce proteinuria and slow the progression of renal proteinuric diseases towards chronic renal failure.^34^

In addition, not only there is a variety of mechanisms that may determine the variation of proteinuria and GFR in posttransplant patients but some of them are difficult to be monitored, such as in cases of noncompliance, or instrumental clinical investigations, that cannot be translated into numerical input parameters.

The aim of this work is to implement a FL‐based expert system in order to assess and to follow‐up the transplanted patients due to renal pathology. To this aim, we evaluated how the blood glucose level and the use of immunosuppressive and ACE inhibitor drugs (considered among easily determinable clinical parameters, which change the renal function) can lead to changes in proteinuria and GFR. Different values of these two notable parameters are associated with the various stages of renal failure, and therefore, they allow the characterization of the severity of the renal pathology from which the patient is affected. For this purpose, it was necessary to implement two case studies by fuzzy inference systems (FIS).

The present study can be considered innovative because the CDSS's outputs represent two clinical parameters (level of proteinuria and GFR) of extreme importance for the evaluation of kidney health and easy to read for clinicians. Furthermore, all the rules of inference were carefully agreed with the physicians of the nephrology department. The advantages brought by fuzzy‐based CDSS implemented for the assessment and follow‐up of kidney transplanted patients can be summarized as follows: (a) improvement monitoring of the proteinuria and GFR thus reducing the risk of rejection and the overdose of drugs; (b) improvement of complications control with viewing of alert system easy to read for the patient too; and (c) reduction of costs of care avoiding test duplication and drug excess.^35^

## 2 METHODS

The FL‐based CDSS's implementation is made with the Matlab (MathWorks, Version 2.3.1 R2018a) computing environment that through the FL toolbox provides the tools for analysing, designing, and simulating systems based on FL. The inference process is developed by Mamdani‐type FIS. Due to intuitive nature of the rule bases, Mamdani‐type FIS is widely used for decision support application, as in the present work where the rules are determined starting from the analysis of the data and, obviously, from a previous clinic knowledge of the subject.^36^

The parameters chosen for the evaluation of renal functionality are proteinuria (which expresses the presence of an abnormal amount of protein in the urine) and the glomerular filtration rate or GFR (which is the amount of filtrate formed by both kidneys in 1 minute). This choice is in accordance with National American Kidney Foundation of Nephrology and Kidney Disease Improving Global Outcomes (KDIGO)^37^ clinical guidelines on the care of the kidney transplant recipient that recommends to measure proteinuria and GFR.^38^

In the two case studies, clinical input parameters are chosen to evaluate the variation of proteinuria. The glycaemia level,^39^ the blood level of the m‐Tor inhibitor,^40^ and the decreasing in the ACE‐inhibitor dosage are known for their influence in proteinuria alterations. Similarly, the clinical input parameters chosen to evaluate the variation of GFR are the glycaemia,^41^ the dosage of calcineurin‐inhibitor,^42^ and the increase in dose of ACE‐inhibitor drug.^43^

The statistical sample is selected through the experimental data provided by the Department of Nephrology of the Hospital Policlinico of University of Study of Naples Federico II. The department activities are mainly aimed at carrying out renal de novo transplants and the follow‐up of patients with renal transplantation. For the follow‐up of patients with renal transplantation, numerous and repeated annual accesses to the structure are required. The complete patient set is made up of 855 units in posttransplant follow‐up, and they are provided by the Department of Nephrology.

In order to select the statistical sample of two case studies, these inclusion/exclusion criteria were followed:

- *The time elapsed since the transplant between 2 and 6 years*, the upper limit of 6 years allows to include the largest number of cases, instead cases in the bottom to 2 years after transplantation were excluded for which it would have been premature to detect the side effects associated with immunosuppressive therapy.
- *The age range of 30 to 60 years* of both genders, the upper limit is determined by the fact that over 60 years the probability of alteration of the clinical parameters under examination for physiological ageing increases, while under 30 years, there are few cases that would have led to a statistical inhomogeneity.
- *Exclusions*:
- Patients who lacked the clinical data of proteinuria in the 24 hours and of the sirolimus blood level were excluded.
- Similarly patients who did not take the cyclosporine and ACE‐inhibitor drugs were excluded.
- Patients were also excluded for medical considerations that showed his/her health strongly influenced by other factors different from those of interest.
- Patients belonging to the trial set (that allowed the definition of the rules of FIS) were excluded.

The inference rules in both case studies are defined starting from a set of patients with the help of the physicians. Similarly, the fuzzy set of input and output variables have been defined. Four different FISs were implemented according to the needs of physicians to monitor directly the effects of individual drugs on the variation of proteinuria and GFR. Once the system is defined, its accuracy is assessed in assigning the patient the level of risk based on proteinuria and GFR values.

The fuzzification of the inputs has been here achieved by using triangular and trapezoidal membership functions defined in accordance with threshold values provided by medical researcher and clinicians of the Department of Nephrology of the Hospital Policlinico of University of Study of Naples Federico II. The membership functions can be of different shape, but trapezoidal and triangular membership functions are most frequently successfully used. Indeed, some studies provide a based theoretical explanation for this choice because the interval interpolation function usually has the same form as interpolation corresponding to the trapezoidal membership functions.^44^

The overlap extent of the membership functions has been agreed with the physicians taking into account the narrow ranges of the different clinical parameters that are usually used for the evaluation of kidney health.

### 2.1 Knowledge representation

The design of a fuzzy inferential system (FIS) requires, first of all, the definition of the domain knowledge in cooperation with clinical experts by means of interviews, questionnaires, and observation of their day‐by‐day clinical practice.^21^ The domain of knowledge embedded into the decision mechanism of the system has been described in terms of linguistic variables, linguistic values, and membership functions. A linguistic variable is a variable whose values are words or sentences in a natural or artificial language that can be used to ease a gradual transition between states, so as to naturally.

Definition 1 Linguistic variable.^45^ A linguistic variable (also named fuzzy variable) can be characterized by a quintuple (L; F(L);U; R; M) in which L is the name of the variable; F(L) is the term‐set of L, that is, the collection of its linguistic values; U is a universe of discourse; R is a syntactic rule which generates the terms in F(L); and M is a semantic rule which associates to every linguistic value X its meaning, M(X), where M(X) denotes a fuzzy subset of U.

Definition 2 Fuzzy variable.^45^ A fuzzy variable is characterized by a triple (L;U; F(L; u)), in which L is the name of the variable; U is a universe of discourse (finite or infinite set); u is a generic name for the elements of U; and F(L; u) is a fuzzy subset of U which represents a fuzzy restriction on the values of u imposed by L. F(L; u) will be referred to as the restriction on u or the restriction imposed by L. The assignment equation for L has the form

$$x=u:F\;\left(L\right)$$

and represents an assignment of a value u to x subject to the restriction F(L).

In the universe of discourse U, a fuzzy set F(L; u) is characterized by a membership function μ(F) that assigns a membership value to elements u, within a predefined range of U, as follows: F = {(u; μ_F_)|u U and μ_F_: U ➔ [0; 1]}. In practice, a membership function is a curve that defines how each element in the input space is mapped to a membership value (or degree of membership) between 0 and 1.

In order to grant a simple interpretation of the knowledge modelled via linguistic variables, linguistic values and membership functions have been designed following the approach presented by Gariabaldi et al.^46^

To perform the fuzzy inference, the knowledge about the medical decision making has been formalized in terms of fuzzy “if‐then rules” relying on the structure defined for the domain of knowledge. In so doing, fuzzy inference relies on rules, defined as conditional statements written in the form “if antecedent then consequent,” where antecedent is a fuzzy‐logic expression composed of one or more simple fuzzy expressions connected by fuzzy operators, and consequent is an expression that assigns linguistic values to the output variables.^21^ Indeed, FL provides a tool that enables to approximate an inference process, ie, the mental process by which human reach a conclusion based on specific evidence.

### 2.2 Knowledge reasoning

To create the inferential engine, for the evaluation of some clinical aspect related to the patients' status, all clinical variables have been linked in a Mamdani‐style FIS according to different rules and membership functions.^47^, ^48^ The Mamdani scheme is a type of fuzzy relational model where each rule is represented by an “if antecedent then consequent” relationship. Mamdani method is widely accepted for capturing expert knowledge. It allows us to describe the expertise in more intuitive, more human‐like manner.^49^ In the following, the Mamdani method is described, and the basic knowledge is implemented into the system. At this stage of the implementation of the fuzzy inference engine, we refer to a multi‐inputs single‐output decision model.

Definition 3 Given m “if antecedent then consequent” fuzzy rules R = {R_1_; : : : ;R_m_}, with n continuous input variables u_i_, i = 1; : : : ; n, and the output variable y, the formulation of the fuzzy rules is defined as follows:

$$\text{if}\left(u_{1};A_{1;1}\right)\text{AND}\left(u_{2};A_{1;2}\right)\text{AND}:::\text{AND}\;\left(u_{n};A_{1;n}\right)\text{THEN}\left(y;B_{1}\right)$$

$$\text{if}\left(u_{1};A_{m;1}\right)\text{AND}\left(u_{2};A_{m;2}\right)\text{AND}:::\text{AND}\;\left(u_{n};A_{m;n}\right)\text{THEN}\left(y;B_{m}\right)$$

where u_i_ are the input variables, y is the output variable, and A_ij_ and B_i_ are fuzzy sets of the associated universes of discourse. Now, to perform inference, the first step is to evaluate the antecedent, which involves fuzzyfying the input and applying any necessary fuzzy operators to each rule in R.

Definition 4 Given the information input u = {u_1_; : : : ; u_n_}, the strength level or membership α_i_ of the rule R_i_ is calculated in terms of the degrees of membership μ_Aij_. If the antecedent clause (the if part) is connected with AND, then

$$\mu_{i}\left(u\right)=\min\;\left(\mu_{\mathrm{Ai};1}\left(u_{1}\right);:::;\mu_{\mathrm{Ai};n}\;\left(u_{n}\right)\right).$$

Else if the antecedent clause is connected with OR, then

μ_i_(u) = max(_A_i_; 1(u_1_); : : :; _A_i_; n (u_n_)).

Each fuzzy rule yields a single number that represents the firing strength of that rule. The second step is “implication,” or applying the result of the antecedent to the consequent. Indeed, the strength level is then used to shape the output fuzzy set that represents the consequent part of the rule.

Definition 5 The operator of implication for the rule R_i_ is defined as the shaping of the “consequent” (the output fuzzy set), based on the “antecedent.” The input of the implication process is a single number given by the “antecedent” (ie, _i computed as in Definition 4), and the output is a fuzzy set:

$$\mu_{\mathrm{Bi}}\left(y\right)=\min\;\left(\alpha_{i}\left(u\right);\mu_{\mathrm{Bi}}\left(y\right)\right),$$

where y is the variable that represents the support value of output the membership function μ_Bi_ (·). Now, in order to unify the outputs of all the rules, we need to aggregate the corresponding output fuzzy set into one single composite set. The inputs of the aggregation process are represented by the clipped fuzzy sets obtained by the implication process. The aggregation method we exploited in our application is the max(·) one. Finally, the defuzzification process has been performed starting from the output fuzzy set resulting from the aggregation process.

Definition 6 The operations of defuzzification is computed as the centre of gravity (COG) of the strength levels:

$$\mathrm{COG}\left(y\right)=\frac{\sum_{i=1}^{m}y\mu_{B_{i}}\left(y\right)}{\sum_{i=1}^{m}\mu_{B_{i}}\left(y\right)}.$$

### 2.3 Case study 1

As regards the first case study, the following two systems have been implemented:

#### 2.3.1 ProtFIS

The variation of proteinuria is evaluated by loading in input two parameters: glycaemia and the blood level of the m‐Tor inhibitor.

Based on the above inclusion and exclusion criteria, 63 patients were evaluated, and they are characterized by

- mean proteinuria of 401.78 mg/24 h (physiological range of proteinuria: 100‐4000 mg/24 h) with standard deviation of 424.19 mg/24 h;
- average normalized sample: 0.13 mg/24 h with standard deviation 0.14 mg/24 h;
- average age: 49 years;
- number of patients with diabetes risk: 12.

Associated membership functions to each of the input‐output variables are used.

For the *glycaemia* input variable, the following threshold values are used (reference values used by physicians): normal for values between 70 and 99 mg/dL; impaired for values between 100 and 125 mg/dL; and diabetes for values greater than 126 mg/dL. Starting from these data, three fuzzy sets are named in order good, alarm, and danger.

For blood level of the S*irolimus* (Rapamune) input variable, the fuzzy sets are identified on the information provided by European Medicines Agency,^50^ according to this agency, and with the medical support, this range is split in alarm down (0‐5 ng/mL), sufficient (4‐7 ng/mL), good (6‐12 ng/mL), alarm up (11‐14 ng/mL), and danger (13‐20 ng/mL).

For the *proteinuria* output variable, the following threshold values are used (reference values used by physicians): physiological proteinuria (150 mg/24 h); light proteinuria (150‐500 mg/24 h); moderate proteinuria (500‐1000 mg/24 h); severe proteinuria (1000‐3000 mg/24 h); and nephrotic proteinuria (>3000 mg/24 h). The five trapezoidal and triangular membership functions coupled with these values are in order: good, sufficient, alterate, alarm, and danger.

A central point in FIS implementation consists in determining the inference rules. These are defined based on a set trial and of the clinical opinion that is fundamental in defining the basis of system knowledge. The same weight has been associated with all the rules, ie, 1.

The inference rules used in the system are shown in Table 1.

**Table 1:** Inference rules of ProtFIS

Rule	Glycaemia Level	Sirolimus Level	Proteinuria Level
1	good	good	good
2	danger	danger danger	
3	good	suff	suff
4	alarm	alarm up	alarm
5	alarm	suff	alterate
6	good	alarm up	alterate
7	alarm	good	suff
8	good	alarm down	suff

#### 2.3.2 GfrFIS

The variation of GFR is evaluated by loading two‐parameter inputs, glycaemia, and the dosage of a calcineurin inhibitor.

Based on the above inclusion and exclusion criteria, 103 patients were evaluated, and they are characterized by

- mean GFR of 65.98 mL/min (physiological range of GFR: 0‐130 mL/min) with standard deviation of 15.86 mL/min;
- average normalized sample: 0.51 mL/min with standard deviation 0.12 mL/min;
- average age: 45 years;
- number of patients with diabetes risk: 38.

For glycaemia input variable, the same membership functions of ProtFIS are used.

For dosage of *calcineurin inhibitor* (cyclosporine) input variable, the reference drug is Neoral; also in this case, fuzzy sets are identified on the information provided by European Medicines Agency,^51^ implementing five fuzzy sets to better characterize the physio‐pathological behaviour of patients, highlighting the risk related to the high dosage: alarm down (0‐2 mg/kg), good (1.5‐5 mg/kg), suff (4‐11 mg/kg), alarm up (8‐15 mg/kg) and danger (12‐20 mg/kg).

*GFR* output is made by MDRD equation^52^ using creatinine serum. Usual threshold values (reference values used by physicians) are stage 1: GFR ≥ 90 mL/min; stage 2: 60 ≤ GFR < 90; stage 3: 30 ≤ GFR < 60; stage 4: 15 < GFR < 30; stage 5: GFR < 15 with five fuzzy set named in order: danger, alarm 4, alarm 3, alarm 2, and good.

The inference rules (with same weight unitary) are shown in Table 2:

**Table 2:** Inference rules of GfrFIS

Rule	Glycaemia Level	Cyclosporine Level	GFR Level
1	danger	danger	danger
2	good	danger	alarm III
3	alarm	alarm up	alarm IV
4	good	suff	alarm II
5	good	good	good
6	alarm	good	alarm II
**7**	danger	good	alarm III

### 2.4 Case study 2

In this case:

- For glycaemia input variable, for proteinuria and Gfr output variables, the same membership functions of case study 1 are used.
- For ACE‐inhibitor (Ramipril), the reference drug is Triatec, (recommended dose by European Medicines Agency^53^: 1.25 mg/d, 2.5 mg/d, 5 mg/d, up to a maximum of 10 mg/d). The differences of ACE‐inhibitor dosage in therapy are split in five fuzzy sets by increasingly higher differences according to the following levels: 0 (no difference), 1, 2, 3, and 4 (maximum possible variation).

The two implemented systems are the following:

#### 2.4.1 ProtACE

The variation of proteinuria is evaluated by loading in input two parameters: glycaemia and the decreasing of the dosage of the ACE‐Inhibitor drug.

Based on the above inclusion and exclusion criteria, 70 patients were evaluated and characterized by:

- mean proteinuria of 532.00 mg/24 h (physiological range of proteinuria: 100‐4000 mg/24 h) with standard deviation of 516.00 mg/24 h;
- average normalized sample: 0.15 mg/24 h with standard deviation 0.15 mg/24 h;
- average age: 51 years;
- number of patients with diabetes risk: 13.

The inference rules used in the ProtACE system are shown in Table 3.

**Table 3:** Inference rules of ProtACE

Rule	Glycaemia Level	DiffACE	Proteinuria Level
**1**	‐	2	suff
**2**	good	1	suff
**3**	alarm	1	alterate
**4**	danger	1	alarm
**5**	alarm	3	alarm
**6**	good	3	alterate
**7**	danger	4	danger
**8**	good	0	good

#### 2.4.2 GfrACE

The variation of GFR is evaluated by loading two‐parameter input, glycaemia, and the increase in the dose of the ACE‐inhibitor drug.

By the same inclusion and exclusion criteria, 107 patients were evaluated and characterized by

- mean GFR of 53.54 mL/min (physiological range of GFR: 0‐130 mL/min) with standard deviation of 16.22 mL/min;
- average normalized sample: 0.41 mL/min with standard deviation 0.13 mL/min;
- average age: 48 years;
- number of patients with diabetes risk:16.

The inference rules used in the GfrACE system are shown in Table 4:

**Table 4:** Inference rules of GfrACE

Rule	Glycaemia Level	DiffACE	GFR Level
1	good	2	alarm III
2	alarm	2	alarm III
3	good	1	alarm II
4	alarm	1	alarm II
5	good	3	alarm III
6	danger	3	alarm IV
7	good	4	alarm II
8	good	0	good
9	danger	4	danger

## 3 RESULTS

One of the major advantages of decision analysis models is their ability to rapidly test their assumptions and input data in order to validate the decision model. To evaluate the accuracy of the implemented systems, we have inserted in each of the four FISs the input data of all the selected patients and then the output of each system is subsequently compared with the clinical experimental data respectively of proteinuria and GFR. Following the results obtained for both case studies are reported.

### 3.1 Case study 1

#### 3.1.1 ProtFIS

**Figure 1:**
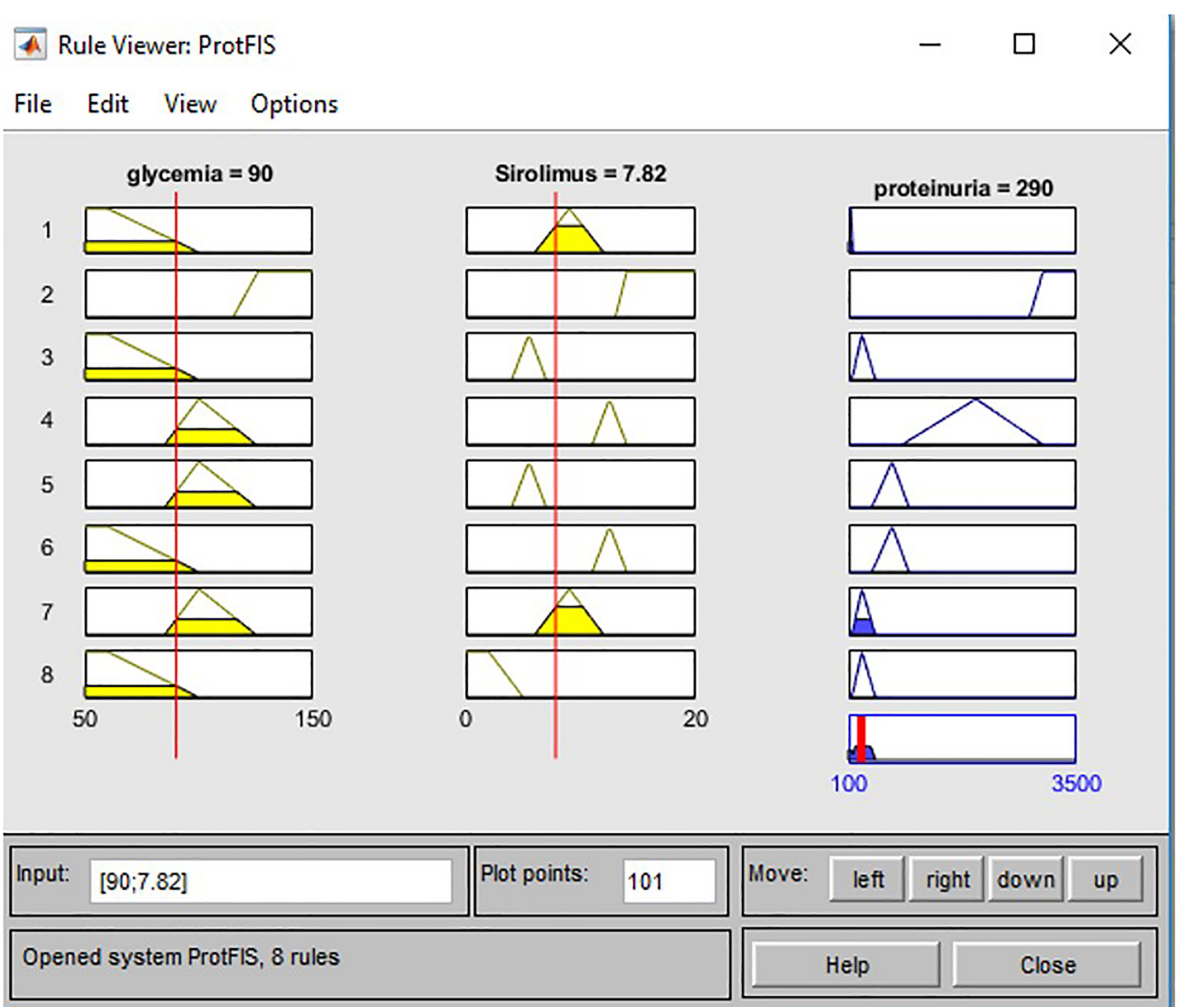
Practical example ProtFIS case study one

By way of example, we report a clinical case related to the ProtFIS illustrated in Figure 1, in which we can see the rule viewer. Each rule is a row of plots, the first two columns show the membership functions referenced by the antecedent, the third column shows the membership functions referenced by the consequent, and the last bottom right plot represents the aggregate decision for the proteinuria, where the red vertical line represents the defuzzified output.

For this patient, the input values are 90 mg/dL glycaemia and 7.82 ng/mL blood level of *Sirolimus*. The proteinuria output is 290 mg/24 h, which is to be compared with the real value provided by the clinical examination of proteinuria equal to 270 mg/24 h. Furthermore, ProtFIS correctly predicts the patient's risk level, placing it in sufficient range, ie, in a light proteinuria level. The overestimation gap of about 5% confirms the good functioning system that tends to place the patient in a band of further protection.

**Figure 2:**
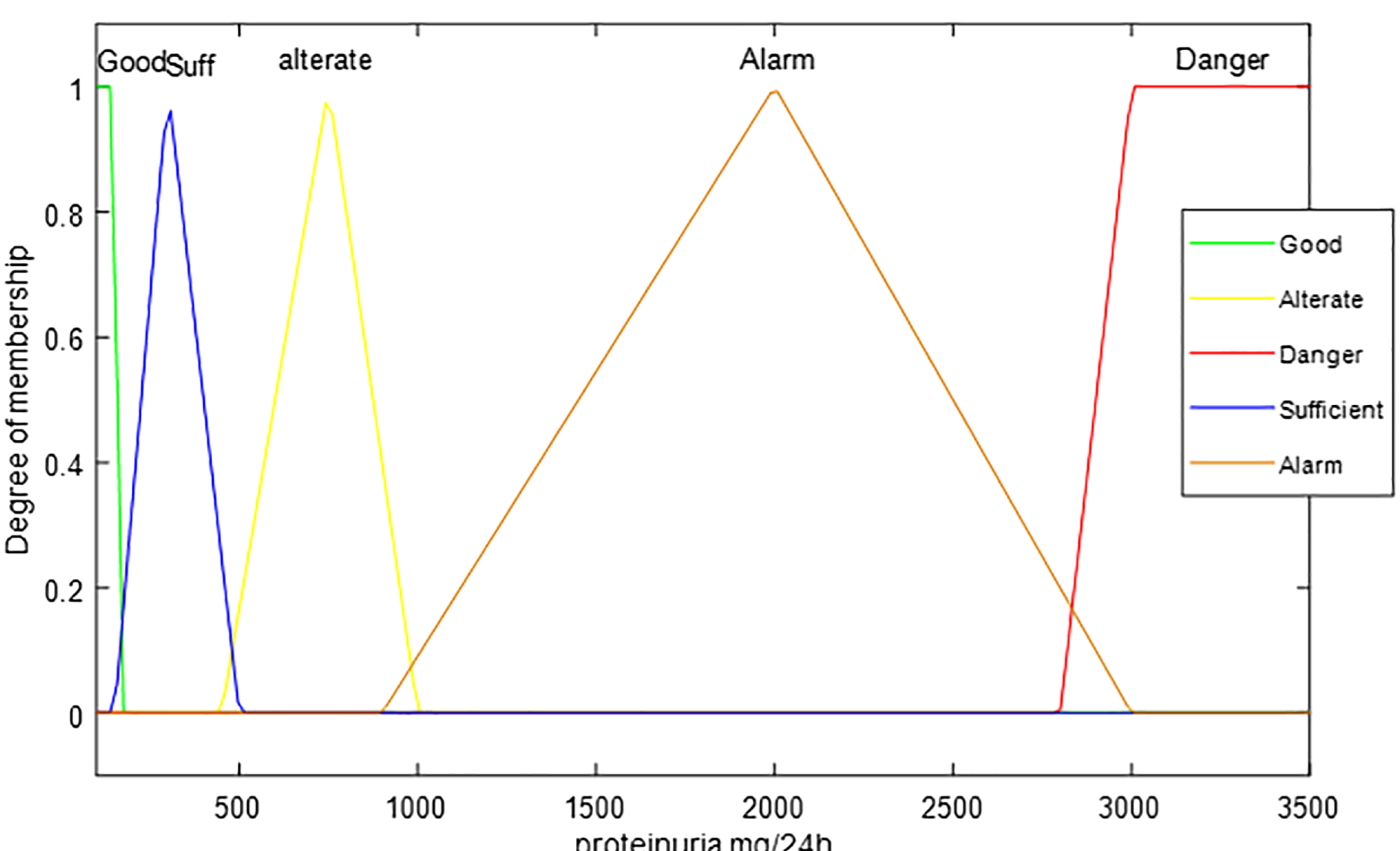
Proteinuria Output case study one

For 63 patients, characterized by a mean proteinuria assessed by the system equal to 495.83 ± 598.92 mg/24 h, the correct evaluations are 57 patients, which is 91%, the errors committed by the system are six out of 63 patients. To make the output of the system easier to interpret, itis made a coloured graph (Figure 2), in which different colours are associated with proteinuria's levels of risk (from green—physiological proteinuria, zero risk—to red—nephrotic proteinuria, high risk), so that physicians can intuitively compare the patient's clinical status with the “Colour Scale” icon.

#### 3.1.2 GfrFIS

**Figure 3:**
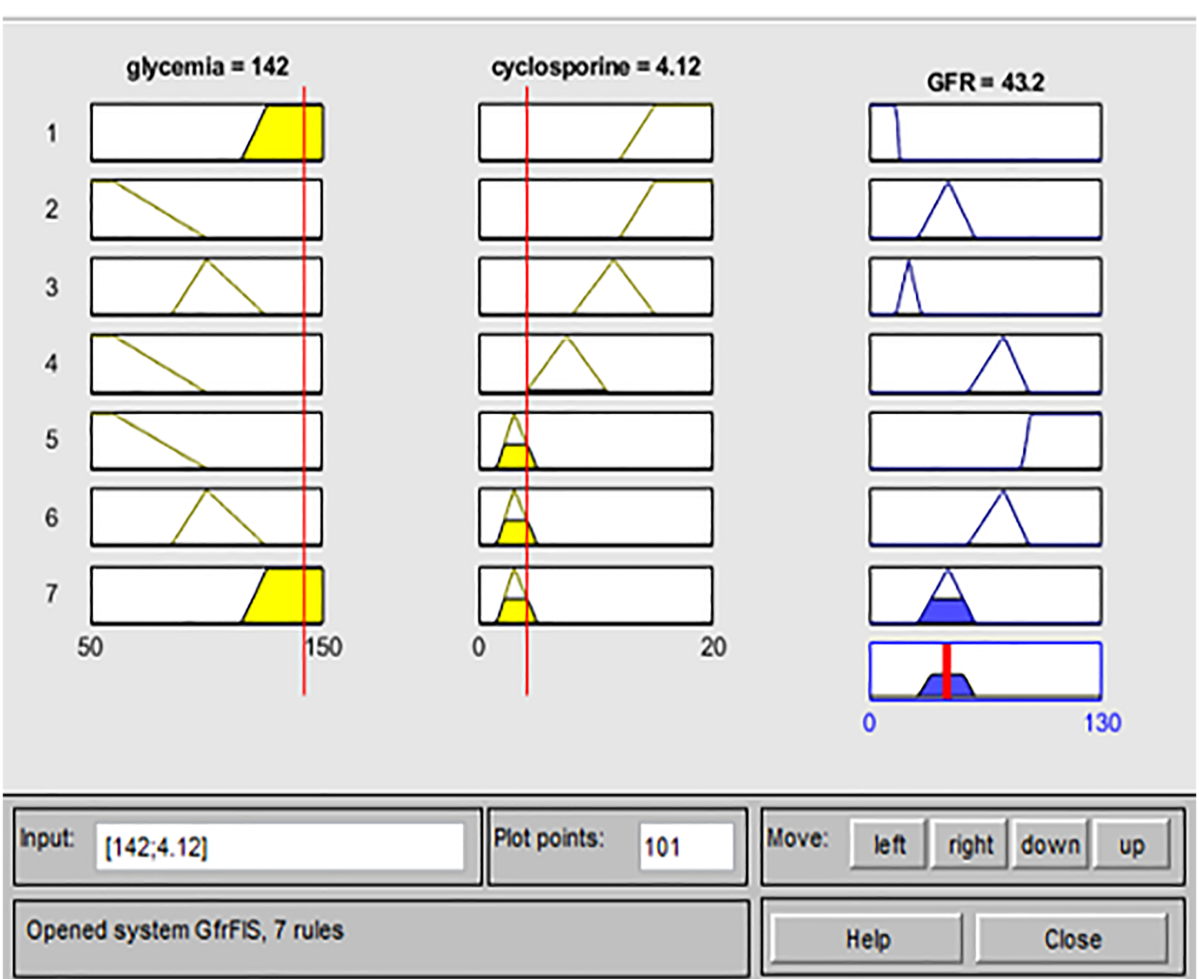
Practical example GfrFIS case study one

Also for this system, we need to validate the model's answer, comparing it with the “true” answer provided by clinical data. For example, we report a clinical case related to the GfrFIS illustrated in Figure 3.

For this patient, the input values are 142 mg/dL of glycaemia and 4.12 mg/kg dosage of a cyclosporine (Neoral). The GFR output is 43.20 mL/min, which is compared with the real value provided by the clinical examination of GFR equal to 51 mL/min. Then, GfrFIS correctly predicted the patient's risk level, placing it in an alarm 3 range corresponding to stage 3. The overestimation gap of about 18% confirms the good functioning system (the estimated value falls in the same risk range provided by the clinical data) that tends to place the patient in a band of further protection.

**Figure 4:**
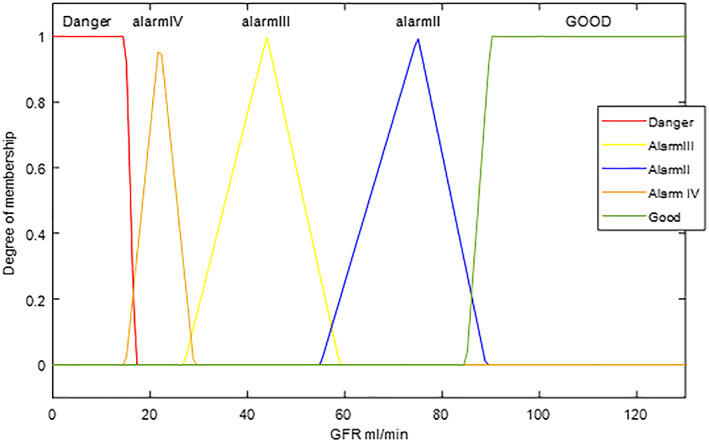
Gfr output case study 1

Out of 102 patients, characterized by a mean GFR assessed by the system equal to 72.60 ± 14.86 mL/min, the correct evaluations are 94 patients, corresponding to 92%, the errors committed by the system are eight out of 102 patients. Even now, the output of the system is graphed by coloured pattern (Figure 4) where it is associated a colour with each level of risk (from green—stage 1 GFR, zero risk—to red—stage 5 GFR, high risk).

### 3.2 Case study 2

#### 3.2.1 ProtACE

**Figure 5:**
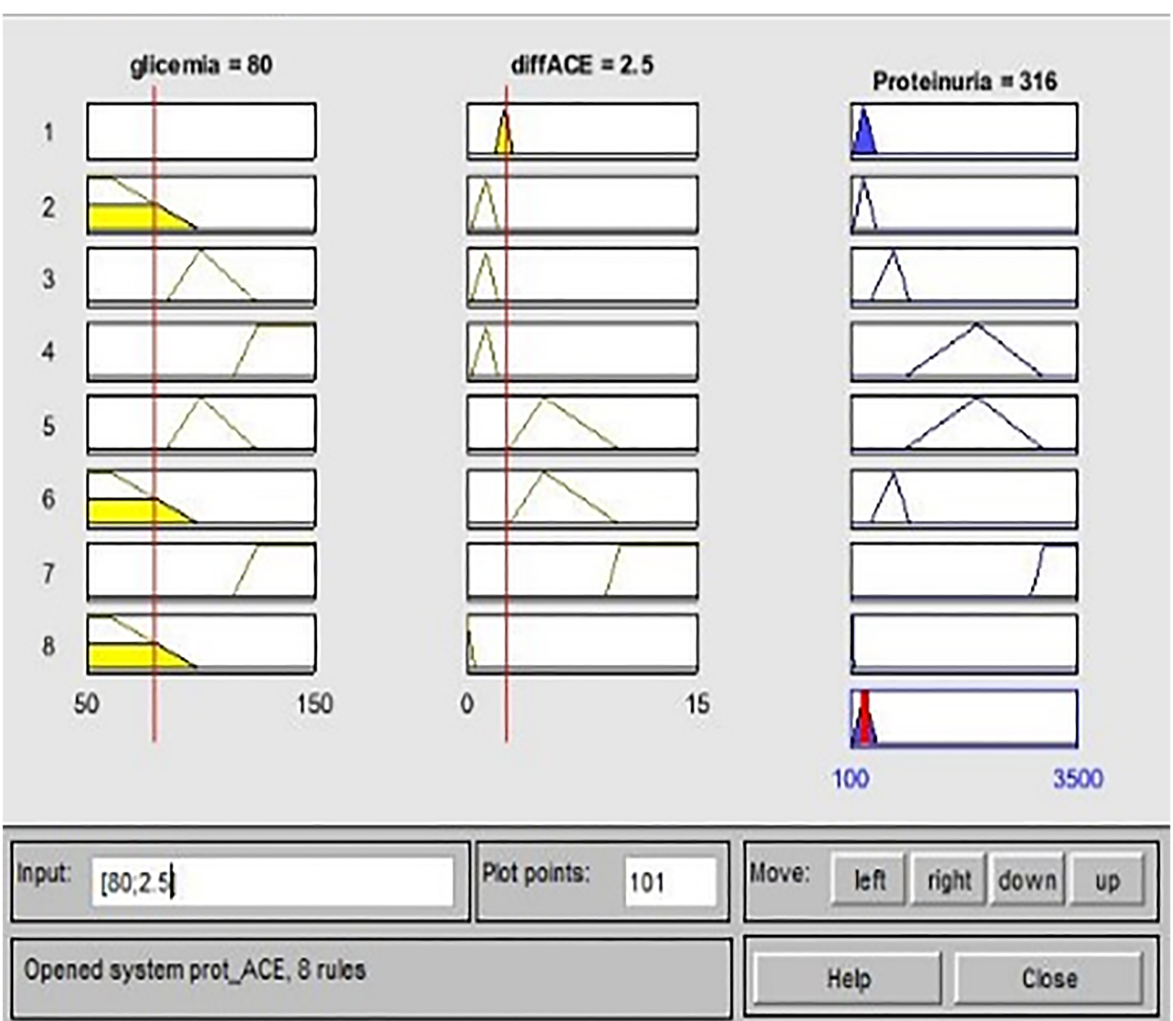
Practical example ProtACE case study 2

For the ProtACE, we show the following clinical case: input 80 mg/dL of glycaemia and 2.50 difference of ACE‐inhibitor dosage (Triatec) (Figure 5). The proteinuria output is 316 mg/24 h, while the clinical examination of proteinuria equal to 315 mg/24 h. So then, ProtACE correctly predicted the patient's risk level, placing it in sufficient range, ie, in a light proteinuria level. The overestimation gap is less than 1% confirming the excellent functioning system.

For 70 patients, characterized by a mean proteinuria assessed by the system equal to 630 mg/24 h and standard deviation of 538 mg/24 h, the correct evaluations are 65, which is 93%.

#### 3.2.2 GfrACE

**Figure 6:**
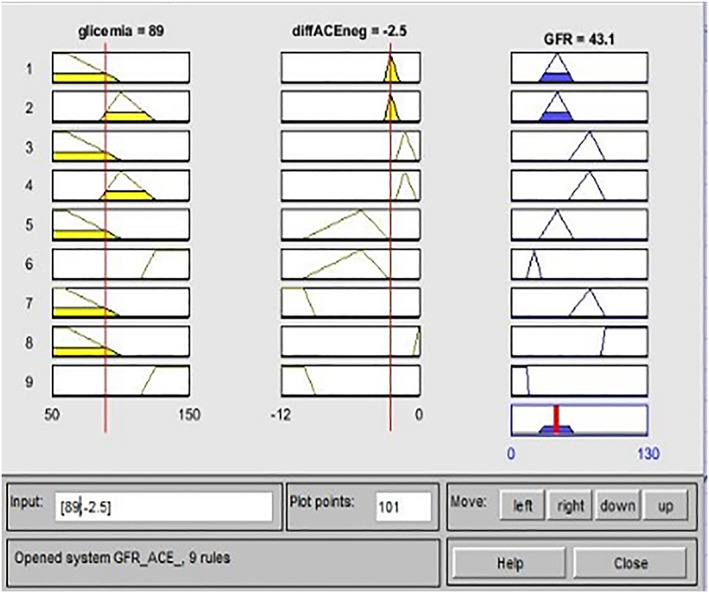
Practical example GfrACE case study 2

The clinical case related to the GfrACE is illustrated in Figure 6 and has the following input values: 89 mg/dL of glycaemia and −2.50 difference of ACE‐inhibitor dosage (Triatec). The GFR output is 43.10 mL/min, which is to be compared with the real value provided by the clinical examination of GFR equal to 53.60 mL/min. So then, GfrFIS correctly predicted the patient's risk level, placing it in the stage 3. The overestimation gap of about 19% confirms the good functioning system, which tends to place the patient in a band of further protection again.

Out of 107 patients, characterized by a mean GFR assessed by the system equal to 51.80 mL/min and standard deviation of 13.90 mL/min, the correct evaluations are 99, corresponding to 93%.

## 4 DISCUSSION AND CONCLUSION

Table 5 shows the results of the performance analysis of the four implemented systems. For all the systems, an accuracy greater than 90% is obtained; this result is fundamental to trust a CDSS, and it is necessary that its reliability can be decidedly high. Nevertheless, the implemented systems in this work do not cover all the possible circumstances that can cause renal damage to posttransplant patients. Indeed, the causes that lead to an increasing in proteinuria or a decreasing in the glomerular filtrate are many and may have more or less serious consequences. In this work, we analysed those factors that have the greatest impact on renal diseases of posttransplant patients; for this, the attention has been focused on the consequences of high blood glucose values and on the use of immunosuppressive and ACE‐inhibitor drugs, these notoriously offer benefits, but can also worsen already critical situations.

**Table 5:** Performance analysis

Systems	ProtFIS, %	GfrFIS, %	ProtACE, %	GfrACE, %
Accuracy	91	92	93	93
Sensitivity	91	96	90	89
Specificity	89	83	94	94
Precision	95	93	91	89
Recall	91	96	90	89
Fmeasure	93	95	90	89
Gmean	90	89	92	91

However, the systems could offer greater efficiency if we consider into account additional input variables related to clinical parameters that could lead to an alteration of proteinuria and GFR. For example, an additional element that could be evaluated is the incidence of the sex of patients; in fact, it is known how it significantly affects the therapeutic response. Including more input and output variables, which no doubt is desirable, would lead to an increase in the number of rules and membership functions; all this, however, could involve a considerable increase in the complexity of the system.

Fuzzy‐based CDSSs implemented for the assessment and follow‐up of kidney‐transplanted patients could improve complications control, using exclusively glucose values, easy to perform, reducing the costs of care and test's duplication, avoiding drugs excess. Moreover, viewing of the alert system, easy to read for both clinicians and patients could stimulate the clinicians to discuss treatment options with patients and consequently make the latter feel more involved in their medical treatment. The goal of the system is obviously to provide effective support, rather than to replace the physician, because, in any case, the physician must filter the information, review the suggestions, and decide how much to consider it before acting. With the gradual maturation of AI health care systems, the CDSSs should play a crucial role in reducing medical errors and in improving the quality of health care and the efficiency of the health care delivery system.

## FUNDING INFORMATION

The are no funding available for this study.

## DISCLOSURE STATEMENT

The authors certify that no financial and/or material support was received for this research and/or the creation of this work.
